# Increased S100A4 expression in the vasculature of human COPD lungs and murine model of smoke-induced emphysema

**DOI:** 10.1186/s12931-015-0284-5

**Published:** 2015-10-20

**Authors:** Sebastian Reimann, Ludger Fink, Jochen Wilhelm, Julia Hoffmann, Mariola Bednorz, Michael Seimetz, Isabel Dessureault, Roger Troesser, Bahil Ghanim, Walter Klepetko, Werner Seeger, Norbert Weissmann, Grazyna Kwapiszewska

**Affiliations:** Excellence Cluster Cardio-Pulmonary System, Universities of Giessen and Marburg Lung Center, Member of the German Center for Lung Research (DZL), Justus-Liebig-University, Giessen, Germany; Institute of Pathology and Cytology, UEGP, Forsthausstrasse 1, 35578 Wetzlar, Germany; Ludwig Boltzmann Institute for Lung Vascular Research, Graz, Austria; Department of Thoracic Surgery, Division of Surgery, Medical University Vienna, Vienna, Austria

**Keywords:** COPD, Hypoxia-inducible factor, Pulmonary hypertension, Smooth muscle cell, S100A4, RAGE, Vascular remodeling

## Abstract

**Background:**

Chronic obstructive lung disease (COPD) is a common cause of death in industrialized countries often induced by exposure to tobacco smoke. A substantial number of patients with COPD also suffer from pulmonary hypertension that may be caused by hypoxia or other hypoxia-independent stimuli - inducing pulmonary vascular remodeling. The Ca^2+^ binding protein, S100A4 is known to play a role in non-COPD-driven vascular remodeling of intrapulmonary arteries. Therefore, we have investigated the potential involvement of S100A4 in COPD induced vascular remodeling.

**Methods:**

Lung tissue was obtained from explanted lungs of five COPD patients and five non-transplanted donor lungs. Additionally, mice lungs of a tobacco-smoke-induced lung emphysema model (exposure for 3 and 8 month) and controls were investigated. Real-time RT-PCR analysis of S100A4 and RAGE mRNA was performed from laser-microdissected intrapulmonary arteries. S100A4 immunohistochemistry was semi-quantitatively evaluated. Mobility shift assay and siRNA knock-down were used to prove hypoxia responsive elements (HRE) and HIF binding within the S100A4 promoter.

**Results:**

Laser-microdissection in combination with real-time PCR analysis revealed higher expression of S100A4 mRNA in intrapulmonary arteries of COPD patients compared to donors. These findings were mirrored by semi-quantitative analysis of S100A4 immunostaining. Analogous to human lungs, in mice with tobacco-smoke-induced emphysema an up-regulation of S100A4 mRNA and protein was observed in intrapulmonary arteries. Putative HREs could be identified in the promoter region of the human S100A4 gene and their functionality was confirmed by mobility shift assay. Knock-down of HIF1/2 by siRNA attenuated hypoxia-dependent increase in S100A4 mRNA levels in human primary pulmonary artery smooth muscle cells. Interestingly, RAGE mRNA expression was enhanced in pulmonary arteries of tobacco-smoke exposed mice but not in pulmonary arteries of COPD patients.

**Conclusions:**

As enhanced S100A4 expression was observed in remodeled intrapulmonary arteries of COPD patients, targeting S100A4 could serve as potential therapeutic option for prevention of vascular remodeling in COPD patients.

**Electronic supplementary material:**

The online version of this article (doi:10.1186/s12931-015-0284-5) contains supplementary material, which is available to authorized users.

## Background

Chronic obstructive pulmonary disease (COPD) is characterized by chronic airflow limitation and pathological changes in the lung and vascular system [1]. COPD encompasses chronic obstructive bronchitis and obstructive lung emphysema, which often interact. Chronic obstructive bronchitis is a chronic airway inflammation with loss of the mucociliary clearance, increased infect-exacerbation rate and bronchus wall instability. In many cases it is caused by smoking [1]. Chronic inflammation, imbalance of protease- and antiprotease activity, and lumen obstruction of small airways lead to destruction and loss of alveolar septa resulting in emphysema. Data from the Global initiative for chronic Obstructive Lung Disease (GOLD-report) estimated that up to 25 % of the adult population aged 40 years or older have COPD [1]. Based on this high prevalence, COPD is a common cause of death in industrialized countries [2]. The soaring burden of COPD is associated with the accumulate incidence of inhalation of tobacco smoke or other noxious particles [1]. Cigarette smoke is one of the highest risk factors known to actively cause the disease [3].

As not all smokers develop clinically significant COPD other factors such as oxidative stress, infection and genetic background contribute to the individual risk [1]. Together these mechanisms lead to the characteristic pathological change in COPD triggered by a chronic lung inflammation [1]. Mucus hypersecretion, extended inflammation and fibrosis in the small airways on the one hand, destruction and loss of alveolar septa on the other hand result in dyspnea and abnormal gas exchange [1]. Later on, these pathophysiological changes often cause pulmonary hypoxemia and hypercapnia [1, 4]. Additionally, a substantial number of COPD patients also suffer from an at least mild increase of pulmonary arterial pressure [5–7] and more than ten percent show a clinical significant pulmonary hypertension (PH), leading to shorter survival [4, 8–10]. Structural changes in pulmonary vascular remodeling include media hypertrophy, thickening of the intima with reduction of the lumen diameter and muscularization of small non-muscular arterioles [4]. Chronic hypoxia may be a trigger, but the vascular alterations appear already before the onset of respiratory insufficiency. Alternatively, cigarette smoke induced chronic inflammation, increase of reactive oxygen species, vascular shear stress and altered endothelial function may trigger the vascular alterations [4, 11].

S100A4 is a member of the S100 calcium-binding proteins [12]. S100A4 is involved in intra- and extracellular activities such as cell motility, angiogenesis, smooth muscle cell (SMC) migration and proliferation [13–15]. Additionally, a role in epithelial mesenchymal transition has been implicated [16, 17]. Lawrie et al. showed that SMC migration and proliferation depended on an autocrine or paracrine stimulation of the RAGE receptor (advanced glycosylation end product-specific receptor) by S100A4 [14]. Furthermore about 5 % of transgenic mice overexpressing S100A4/Mts1 exhibit the formation of plexiform lesions with intima hyperplasia [18]. Utilizing the hypoxic mouse model of PH we have previously demonstrated a strong up-regulation of S100A4 during hypoxia exposure [19]. Expression of S100A4 was predominately localized to the smooth muscle cells and to neo-muscularized resistance vessels [19]. These data may point to the involvement of S100A4 in vascular remodeling. Therefore, the aim of the present study was to investigate S100A4 in the context of vascular remodeling within chronic obstructive pulmonary disease. The established mouse model of smoke-induced emphysema [20] was utilized to analyze the expression and specific localization of S100A4. Moreover, we investigated S100A4 in lung tissue samples from explanted COPD and non-transplanted donor lungs.

## Methods

### Tobacco smoke exposure of mice

Adult male wild type (WT) C57BL6/J mice (20–22 g) were obtained from Charles River Laboratories, Sulzfeld Germany. All experiments were approved by the governmental ethics committee for animal welfare (Regierungspräsidium Giessen, Germany). In a current study material from the same experimental animals was used as in Seimetz et al. [20]. Briefly, four mice each were randomly assigned to treatment (tobacco smoke exposure) and control (not exposed to smoke). Mice of the treatment groups were exposed to mainstream smoke of 3R4F cigarettes (University of Kentucky, Lexington, KY, USA) in a concentration of 140 mg particulate matter/m^3^ for 6 h/day, 5 days/week for three or eight months. Age matched control groups were kept under identical conditions but without smoke exposure. Morphometric and hemodynamic data from the same animals were shown previously [20]. In brief, these mice developed PH (reflected by increased right ventricular systolic pressure and vascular muscularization) already after 3 months of smoke exposure. In contrast, 8 months mice had not only fully developed PH but also emphysema (as shown by reduced lung function and increased mean linear intercept as well as decreased septal wall thickness). The smoke-exposed mice neither suffered from hypoxemia nor from hypoxia within the chamber during the smoke exposure as reflected by no changes in O_2_ concentration.

### Human tissues

Lung tissues were obtained from explanted lungs of COPD patients who underwent lung transplantation at the Department of Surgery, Division of Thoracic Surgery, Medical University of Vienna, Austria. Explanted lungs were collected at the time of transplantation. The study was approved by the local ethics committees (Vienna, Austria and Giessen, Germany). Tissues from five patients with COPD (3 male, 2 female, mean age: 54.2 years, GOLD-Stage 3–4) and five control subjects of organ donors (3 male, 2 female, mean age: 44.4 years) were used. Gender and age of donors and COPD patients are given in the Additional files 1.

### Laser-assisted microdissection

Intrapulmonary arteries with a diameter of 50–300 μm were microdissected by Microlaser Technology (P.A.L.M., Bernried, Germany) [21, 22]. Summarized information is provided in online data supplement.

### RNA extraction

Total RNA was isolated with the RNeasy Kits (Qiagen, Hilden, Germany). For more details please refer to online data supplement.

### cDNA synthesis

First strand cDNA from microdissected human tissues and mouse lung homogenate was generated as described previously [22]. For more details please refer to online data supplement.

### Relative mRNA quantification by real-time PCR

Gene regulation was analyzed by real-time PCR using the 7900HT Real-Time PCR-System (PE Applied Biosystems, Forster City, USA). Normalized expression levels were measured by ∆c_T_-values as described previously [19]. PBGD and B2M were used as reference genes [22, 23]. Melting curve analysis and gel electrophoresis was performed to confirm the exclusive amplification of the expected PCR product. Reaction mixtures, detailed cycling conditions, and primer sequences are provided in Additional file.

### Immunhistochemistry

Murine cryo sections were incubated with rabbit polyclonal S100A4 antibody (Abcam, Cambridge, UK; 1:500 dilution in Real™ Antibody Diluent (Dako)). Human paraffin sections were incubated with rabbit polyclonal S100A4 antibody (Abcam; 1:700) in 10 % BSA (Sigma-Aldrich). For SMC staining rabbit polyclonal SMC α-actin antibody was used (NeoMarkers, Fermont, USA; 1:350). Anti-RAGE immunohistochemistry was performed on human and mouse paraffin sections (Abcam: 1:500). For detailed information please refer to Additional file 1.

### Semi quantitative analysis of immunhistologically stained tissue sections

Pictures were taken with Discus software (Hilgers, Königswinter, Germany), stored in TIF files and analyzed for staining intensity by Adobe Photoshop® (Adobe System GmbH, Munich, Germany) software. For detailed information please refer to online supplement.

### Hypoxia response element (HRE)

Genomic sequence of human S100A4 was obtained from (http://www.ncbi.nlm.nih.gov/mapview/) and screened for presence of hypoxia response elements (HRE) 5000 bp downstream and upstream from coding sequence. The consensus sequence chosen for HRE was “ACGTGS”, were S can be G or C [24].

### Cell culture

Primary PASMC were isolated from human pulmonary arteries from non-transplantable donor lungs. All experiments were performed with cells in passage three to six and growth arrested by serum deprivation for 24 h. For the investigation of the effect of hypoxia, cells were either exposed to 1 % O_2_ (hypoxia) or to 21 % O_2_ (normoxia) for 24 h. To control for non-specific gene inhibition of the siRNAs used in this study, a universal negative-control siRNA sequence was employed (Ambion, Austin, USA). Cells were transfected with siRNA (100 nM) using the X-treme Gene siRNA Transfection Reagent (Roche, Mannheim, Germany).

### Nuclear extraction

Protein extract from human PASMC was isolated with the NE-PER Nuclear and Cytoplasmic Extraction Reagents (Pierce, Rockford, USA) following the instructions of the user manual.

### Electrophoretic Mobility Shift Assay (EMSA)

EMSA was performed with LightShift Chemiluminescent EMSA Kit (Pierce, USA) following the instructions of the user manual. Reaction mixtures and polyacrylamid gel preparation is provided in the Additional file 1.

### Statistical analysis

**Fig. 1:**
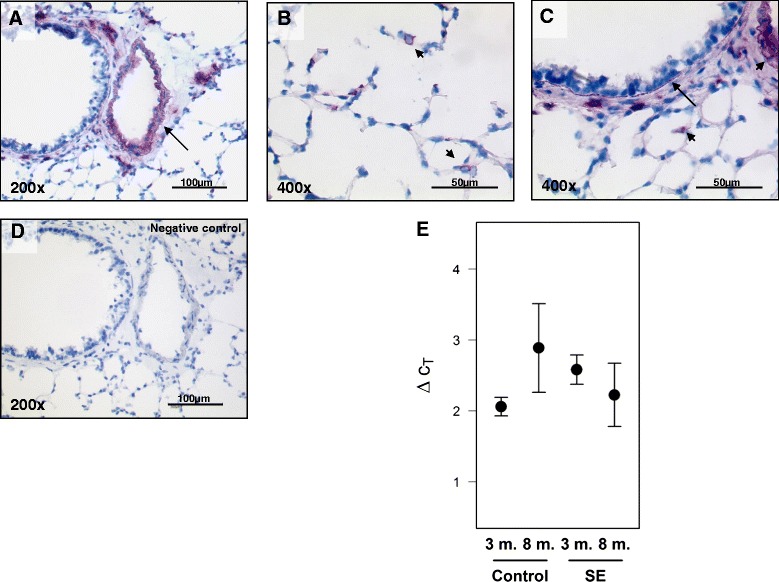
S100A4 expression in mouse lungs after smoke-exposure. Immunohistochemical staining of S100A4 protein in **a** intrapulmonary vessels, **b** mononuclear cells, **c** smooth muscle cells of bronchus. **d** negative control. Bars represent 50 μm and 100 μm. **e** real-time PCR analysis of S100A4 from lung homogenates after three and eight months of smoke exposure compared to three and eight months control animals. 3 m-three months, 8 m-eight months, SE-smoke exposure

**Fig. 2:**
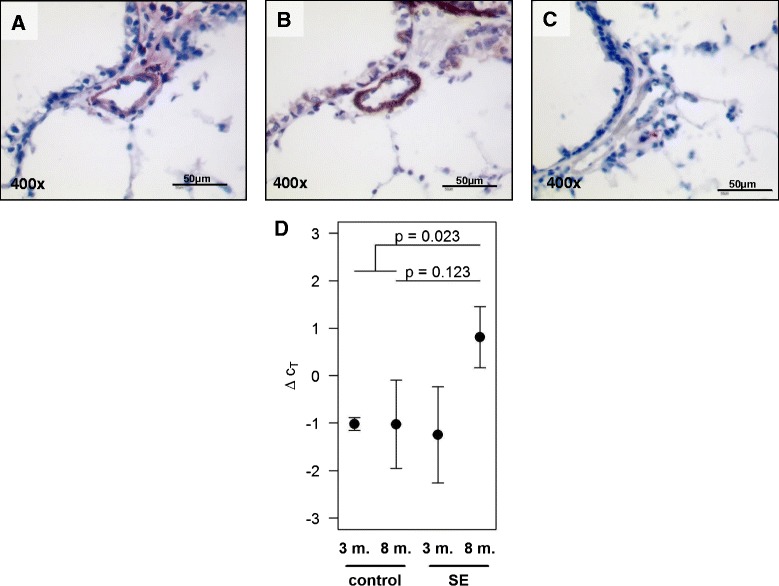
S100A4 localization and mRNA expression in intrapulmonary vessels in mouse lungs after smoke-exposure. Immunohistochemical staining of **a** S100A4, **b** α smooth muscle actin in serial section from mouse lungs, **c** negative control. Bars represent 50 μm. **d** real-time PCR analysis of S100A4 from laser-microdissected arteries after three and eight months of smoke exposure compared to control animals. 3 m-three months, 8 m-eight months, SE-smoke exposure. The two *p*-values indicate the results from the linear hypothesis about difference between SE and controls at eight months tested within a 2-factorial model (*p* = 0.123) and the result from testing the difference between SE at 8 month against the pooled controls (*p* = 0.023)

Values are presented as mean ± SEM. The analyses for statistical significance were calculated with the following tests: Figs. 1e and 2d: two-factorial ANOVA (time × treatment), tested hypotheses: Δct(3 m,control) – Δct(3 m,se) == 0, Δct(8 m,control) – Δct(8 m,se) == 0. Note: neither interaction nor main effects were of interest. Since the data from the microdissected samples did not show any recognizable difference between the two controls (3 and 8 months), the effect after 8 months SE data was additionally estimated using the pooled data of these controls.

**Fig. 3:**
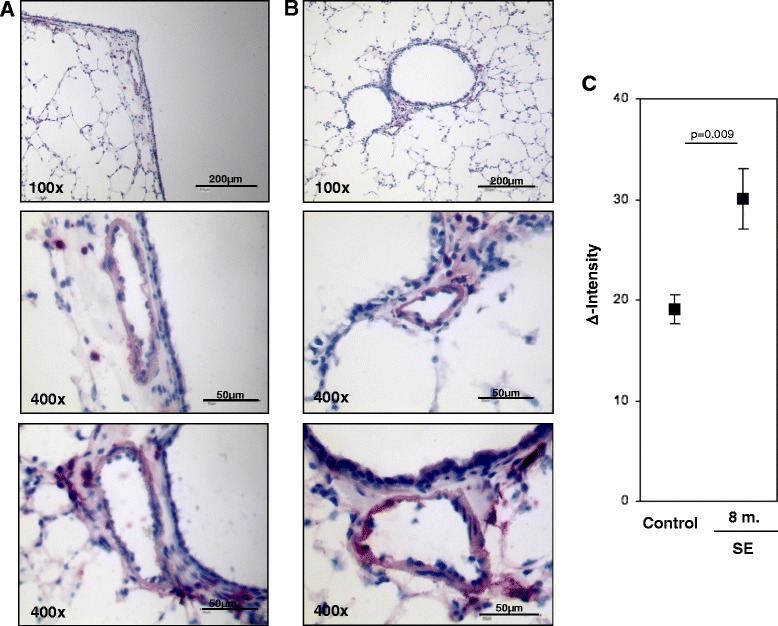
S100A4 protein expression in intrapulmonary vessels in mouse lungs after smoke-exposure. Representative images of intrapulmonary arteries from **a** control animals, **b** smoke exposed animals. Bars represent 50 μm and 200 μm. **c** semi-quantitative analysis of S100A4 protein expression in intrapulmonary arteries. Δ Intensity- color intensity of S100A4 protein after immunohistochemical staining. 8 m-eight months, SE-smoke exposure

**Fig. 4:**
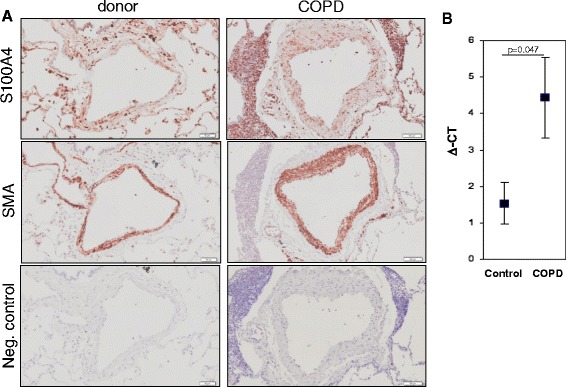
S100A4 localisation and expression in human intrapulmonary arteries. Immonohistochemical staining of S100A4, α smooth muscle actin and negative control in intrapulmonary vessels of COPD patients and donor lungs. Bars represent 100 μm. **b** S100A4 expression in laser-microdissected arteries from donor and COPD lungs

**Fig. 5:**
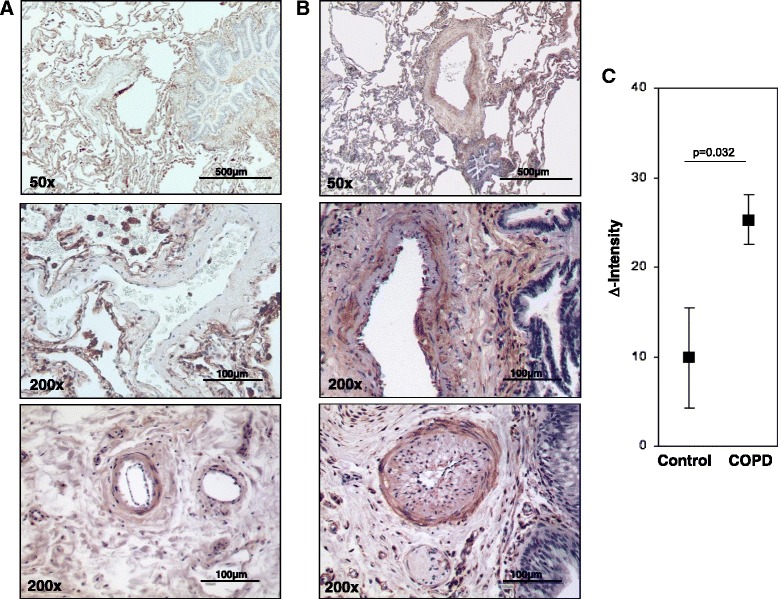
S100A4 protein expression in intrapulmonary vessels of donor and COPD lungs. Representative images of intrapulmonary arteries from **a** control lungs, **b** COPD patients. Bars represent 100 μm and 500 μm. **c** semi-quantitative analysis of S100A4 protein expression in intrapulmonary arteries. Δ Intensity- color intensity of S100A4 protein after immunohistochemical staining

**Fig. 6:**
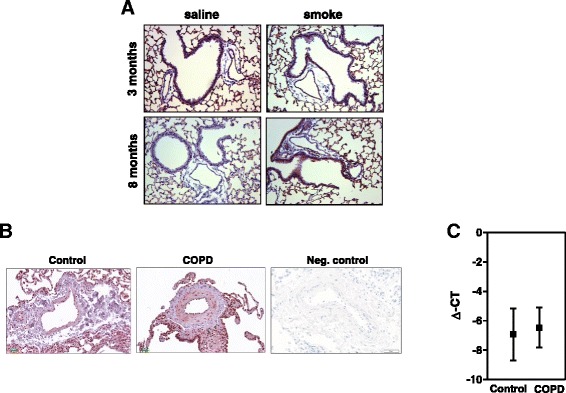
RAGE mRNA and protein expression in murine emphysema model and human COPD. **a** Representative images of intrapulmonary arteries from control and from 3 and 8 month smoke exposed animals. **b** Representative images of intrapulmonary arteries from control and COPD patients. Bars represent 100 μm. **c** RAGE expression in laser-microdissected arteries from donor and COPD lungs

**Fig. 7:**
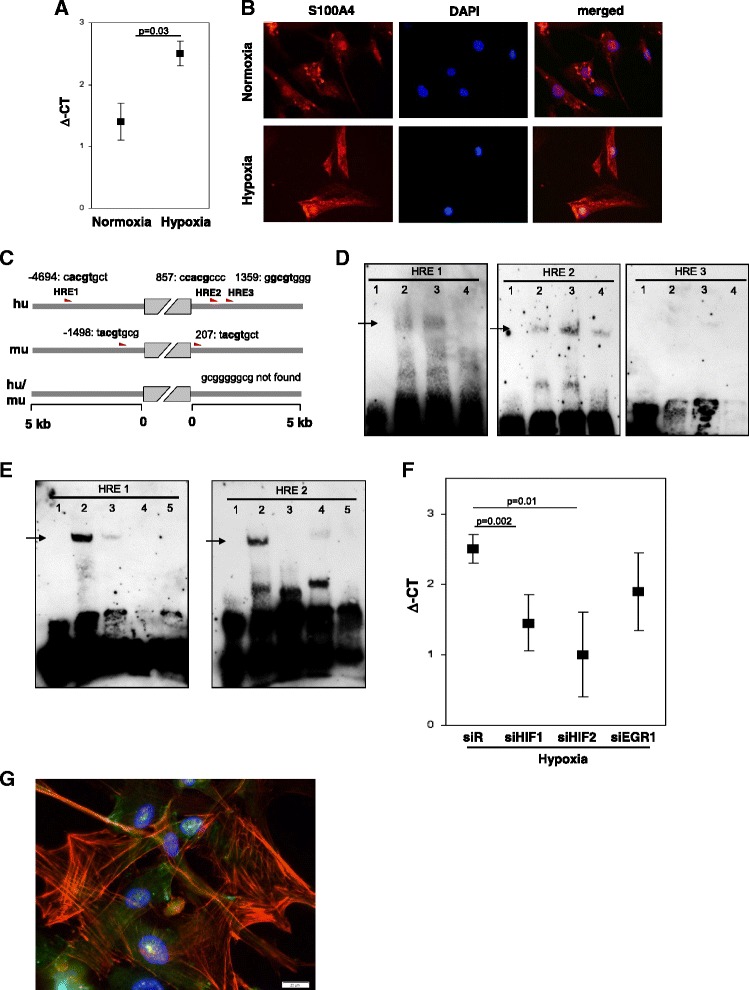
Hypoxia-dependent S100A4 expression in human primary pulmonary arterial cells. **a** Real-time analysis of S100A4 expression in human PASMC after normoxia and hypoxia exposure (24 h). **b** Representative images of S100A4 immunofluorescence labeling in human PASMC after normoxia and hypoxia treatment. **c** Schematic representation of potential HRE in upstream and downstream sequence from S100A4 coding site. **d** EMSA analyses of potential HREs [HRE1:-4694, HRE2:857, HRE3:1359]. Slots were loaded as follows: 1: Labeled Probe only, 2: Labeled Probe and Nuclear extract (24 h normoxia), 3 Labeled Probe, Nuclear extract (24 h hypoxia), 4: Labeled Probe, Nuclear extract (24 h hypoxia) and Competitor. **e** Supershift analysis: 1: Labeled Probe only, 2: Labeled Probe and Nuclear extract (24 h hypoxia), 3 Labeled Probe, Nuclear extract (24 h hypoxia) and Competitor, 4: Labeled Probe, Nuclear extract (24 h hypoxia) and Antibody against HIF-1, 5: Labeled Probe, Nuclear extract (24 h hypoxia) and Antibody against HIF-2. Arrows represent specific band. **f** Real-time analysis of S100A4 expression after pretreatment of human PASMC with siRNA against HIF1, HIF2 or EGR1. ΔCt- relative expression of S100A4 to reference gene. siR- siRandom. **g** Immunofluorescence labeling of S100A4, SMC-actin and DAPI

Figs. 3c, 4c, 5c, 6c, 7a, Two-sample *t*-test (two-sided). Fig. 7f Dunnett’s multiple-to-one tests against siR (two-sided).

## Results

### S100A4 localization and expression in the tobacco-smoke induced murine model of emphysema

Initially we examined the pattern of S100A4 expression in mouse lungs after three and eight months of smoke exposure by immunohistochemical staining. Lungs from both treated and control mice, exhibited similar localization. Strong staining was observed in intrapulmonary arteries, in mononuclear cells located in alveolar septa, as well as in the thin muscular layer of bronchi (Fig. 1a–d). Furthermore we quantified the mRNA expression of S100A4 in lung homogenates of the treated mice compared to respective control animals. As assessed by real-time PCR analysis, there was no significant change in the mRNA level of S100A4 in lung homogenates after three months (2.58 ± 0.21; *p* = 0.06) or eight months of smoke exposure (2.22 ± 0.45; *p* = 0.44) compared to control animals (2.06 ± 0.13 after 3 months; 2.89 ± 0.69 after 8 months) (Fig. 1e).

### S100A4 expression of intrapulmonary arteries in the tobacco-smoke induced murine model of emphysema

In all lungs, specific immune reactivity for the S100A4 protein was observed predominantly in the vascular compartment (Fig. 2a). Immunostaining of adjacent sections with α-smooth muscle actin (SMA) further showed that S100A4 expression occurred in a similar spatial pattern, supporting its predominant expression in the media layer of the arterial wall (Fig. 2b). As strong immunoreactivity of S100A4 was observed in the vasculature wall, we performed laser-microdissection of intrapulmonary arteries with a diameter of 50–300 μm for specific analysis of this compartment. No significant regulation of S100A4 mRNA was detected in microdissected arteries after three months of smoke exposure (−1.25 ± 1.01; *p* = 0.89). In contrast a very strong increase in S100A4 mRNA was observed after eight months of smoke exposure. The effect of smoke exposure was estimated as 1.8 with a 95 % confidence interval from −0.6 to +4.3 (*p* = 0.123). The large uncertainty was due to the very small sample size of 8-month controls (*n* = 3). Since the mean values of the controls were very similar at both time points (−1.023 and −1.025 for 3 and 8 months, respectively), the analysis was repeated with pooled data for the controls, leading to an estimated effect of 1.8 with a 95 % confidence interval from +0.31 to +3.36 (*p* = 0.023) (Fig. 2d).

### Semi-quantitative S100A4 protein analysis in pulmonary arteries

The expression of S100A4 protein was further evaluated by immunohistochemical analysis of cryo sections from four lungs after eight months of smoke inhalation and eight control lungs. Similar to small resistant vessels, S100A4 was also expressed in vessels with a diameter >150 μm. To ensure representative measurements, ten vessels with a diameter between 25–250 μm from each animal were randomly selected. Semi-quantitative analysis of the color intensities revealed increased S100A4 protein levels in the vasculature wall in the animals after eight months of smoke exposure (mean Δintensity ± sem: 30.105 ± 2.99) as compared to controls (mean Δintensity ± sem: 19.11 ± 1.43; *p* = 0.009) (Fig. 3a–c).

### S100A4 localization and expression in chronic obstructive pulmonary disease

In addition to the experimental emphysema mouse model, we examined the localization and expression of S100A4 in patients suffering from COPD. In the human lung sections, S100A4 was predominantly expressed in the vessels (Fig. 4a–c and Fig. 5ab). Immunoreactivity was most intense within the tunica media of pulmonary arteries. S100A4 protein was not only located in the media of occlusive arteries but also in neo-muscularized vessels with a diameter below 50 μm. S100A4 expression exhibited a similar spatial pattern to α-SMA supporting its predominant expression in pulmonary SMC (Fig. 4a–c). Consequently, laser-assisted microdissection of intrapulmonary arteries in combination with real-time PCR was performed for a compartment-specific analysis of mRNA expression. S100A4 mRNA expression was higher in arteries from the lungs of COPD patients (mean ΔCt ± sem: 4.44 ± 0.56) as compared to healthy donors lungs (mean ΔCt ± sem: 1.54 ± 1.1; *p* = 0.047) (Fig. 4d). Furthermore, semi-quantitative immunohistochemical analysis revealed an increased S100A4 protein level in intrapulmonary arteries from COPD-patients (mean Δintensity ± sem: 25.29 ± 2.8) in contrast to healthy control lungs (mean Δintensity ± sem: 9.9 ± 5.6; *p* = 0.032) (Fig. 5c).

### RAGE expression in tobacco-smoke induced murine model of emphysema and in human COPD lung tissue

Lawrie et al. indicated that RAGE could be the receptor for S100A4 [14]. Therefore, we examined whether the expression of RAGE was changed in murine model of emphysema and human COPD lungs. In the mouse lung sections, RAGE positivity was ubiquitously present throughout the tissue (Fig. 6a). Laser-microdissection of intrapulmonary arteries followed by microarray analysis (Agilent 4 × 44 k Human Genome Microarrays. Catalog no. G4112F, design-ID 014850); *n* = 3 animals each, data not shown in detail) revealed significantly increased levels of RAGE mRNA (3 months smoke exposure: Log fold change: 4.9, *p* = 0.000003 and 8 months smoke exposure: Log fold change: 5.2, *p* = 0.0000006). In contrast, although high positivity of RAGE was observed in intrapulmonary arteries of COPD lungs (Fig. 6b), the mRNA levels were comparable to controls (Fig. 6c).

### Hypoxia-dependent S100A4 regulation in primary PASMC

Immunofluorescence analysis of S100A4 in human primary PASMC revealed that S100A4 was localized to the cytoplasm and nucleus (Fig. 7b). As almost all end stage COPD patients suffer from hypoxemia [25] we examined whether hypoxia can influence S100A4 regulation. S100A4 mRNA expression was increased in PASMC cultured under hypoxic (1 % O_2_) conditions for 24 h (mean ΔCt ± sem: 2.45 ± 0.2) compared to normoxia (21 % O_2_) (mean ΔCt ± sem: 1.4 ± 0.3; *p* = 0.028) (Fig. 7a). Due to this hypoxia-dependent regulation of S100A4, the murine and human S100A4 genes were screened for the presence of putative hypoxia response elements (HREs) at a distance of 5 kb upstream and downstream of the coding region. These HREs are of particular interest, as their presence is required for the regulation of mRNA expression by the hypoxia-inducible factors HIF-1 and HIF-2. Importantly, HIF-1/2 are not only stabilized under hypoxic conditions but also under normoxia in the presence of ROS [26] that further underlines the importance of this transcription factor in gene regulation in conditions such as tobacco smoke exposure. Computational analysis of the S100A4 gene was employed to detect the consensus sequence of the HREs (ACGTGS, with S being either G or C). Three and two consensus sequences were found in the sense strand of both, human and murine promoter, respectively. The HREs were located at positions −1498 (t**acgt**gcg), 207 (t**acgt**gct) in murine and at −4694 [HRE-1] (c**acgt**gct), 857 [HRE-2] (c**cacg**ccc) and 1359 [HRE-3] (g**gcgt**ggg) in human sequence (Fig. 7c). The putative HREs [1–3] in S100A4 non-coding sequence were analyzed by EMSA utilizing human PASMC kept under normoxia or hypoxia (1 % O2) for 24 h. EMSA revealed a specific binding of HIF to human HREs only at position −4694 and 857 (Fig. 7d). Preincubation with HIF1α or HIF2α antibodies led to disappearance of the shift band, which might be a result of blocking DNA-protein-complex by HIF antibody (Fig. 7e). The HIF-dependent S100A4 expression regulation was further investigated by siRNA studies. Pretreatment of human PASMC with siRNA against HIF-1α or HIF-2α attenuated hypoxia-dependent up-regulation of S100A4 (Fig. 7d). As the promoter of S100A4 does not contain any Egr-1 consensus sequences (gcgggggcg), siRNA against Egr-1 served as a negative control. As depicted in Fig. 7d, siEgr1 did not influence hypoxia-dependent regulation of S100A4. The overlay of S100A4 and SMC-actin immunofluorescence confirmed co-expression of both proteins in the same smooth muscle cell (Fig. 7g).

## Discussion

In COPD, PH is one of the most frequent complications associated with shorter survival rates [4, 25]. PH has been recognized as one of the predictive factors that is connected with worse clinical outcomes [1, 7, 27]. However, the pathophysiological origin of PH in COPD is mostly unknown. One of the main pathophysiological changes is the remodeling of pulmonary arteries [27]. Previous investigations have shown that vascular alterations and PH precede lung emphysema development in human and animals [20, 28, 29]. Several studies have indicated a possible role of S100A4, a member of the calcium binding proteins, in non-COPD associated forms of PH. Children with congenital heart disease show PH with pulmonary vascular lesions and an increase in S100A4 expression [18]. Furthermore, it has been hypothesized that the S100A4 protein regulates the motility of cells by controlling the cytoskeletal dynamics and promoting SMC proliferation [30]. In the current study we have shown an up-regulation of S100A4 mRNA in microdissected intrapulmonary arteries from explanted end-stage COPD patients. High S100A4 expression was observed in pulmonary arteries not only located in the media of occlusive arteries but also in neo-muscularized vessels with a diameter of ~50 μm. These findings may point to a role of S100A4 in vascular remodeling.

Patients with end-stage COPD and cor pulmonale have a frequent occurrence of hypoxemia in the lungs [25]. Post-mortem studies and analysis of explant lungs have shown the accumulation of smooth muscle cells in the media layer, together with thickening and fibrosis of the intima in pulmonary muscular arteries in these patients [4, 31]. Hypoxic conditions induce up-regulation of S100A4 that is correlated with a thickening of the media layer in intrapulmonary arteries [19]. Up-regulation under hypoxia is induced via HIF transcription factors. While HIF-1β is constitutively expressed in many cell types, HIF-1α is rapidly degraded by ubiquitin-proteasome system under normoxia. Under hypoxic conditions, HIF-1α is stabilized and can form heterodimers with HIF-1β. Upon translocation to the nucleus, they bind to HREs in the promoter region of the target genes [11, 32]. We and others have demonstrated that S100A4 transcription is enhanced via HIF stabilization and promoter binding [33]. However, recent studies point out that remodeling is not exclusive to patients with advanced disease as it has also been shown in patients with mild COPD who do not exhibit hypoxaemia [28, 29, 34]. Barberà et al. [4] showed vascular remodeling in smokers with normal lung function or mild COPD-patients without hypoxia indicating that other mechanisms than hypoxaemia are causative for PH in COPD in earlier stages [4].

Cigarette smoking is one of the most important risk factors for COPD [1, 3]. There are few animal models which imitate the pathological changes seen in COPD [35]. One of the most accepted models is the exposure of animals to cigarette smoke, which appears to be one of the best approximations to the human disease [35]. Typical pathological changes of the human COPD such as emphysema, and PH are also seen in this model [20, 34]. Similar to our observations in sections from COPD patients, mice exposed to 8 month cigarette smoke showed considerable up-regulation of S100A4 mRNA in intrapulmonary arteries, a time-point which largely reflects the characteristics of human COPD. Furthermore, strong immune reactivity for S100A4 protein in the vascular compartment, especially in the media layer was observed. As we have shown previously, in our model mice exposed to cigarette smoke do not suffer from hypoxemia [20], the hypoxia stimulus for induction of S100A4 expression can be excluded in this model. This supports the hypothesis that S100A4 may be involved in early vascular remodeling even in non-hypoxic, mild COPD-stages. But what is the mechanism behind the up-regulation of S100A4 in absence of hypoxia? We could show that human S100A4 gene contains functional putative HREs in its promoters and that siRNA-knockdown of HIF-1/2 decreases S100A4 expression in human PASMC. It is well recognized that HIF-1/2 is not only stabilized under hypoxic conditions but also under normoxia in the presence of ROS [26, 36–38]. Regarding the origin of ROS, Guo et al. showed that nicotine, a major component of cigarette smoke, induces HIF-1α expression via mitochondrial reactive oxygen species in human non-small cell lung cancer cells [38]. In addition, ROS may be derived directly from mainstream smoke that exist mainly in the gaseous phase [26, 38–40]. ROS may activate Erk-5 (BMK-1) via c-Src kinase, which is thought to be an activator kinase of HIF-1α [26]. Alternatively, direct induction of HIF1α by nicotine via acetylcholine receptor-mediated signaling cascades, including the Ca^2+^/calmodulin, c-Src, protein kinase C, phosphatidylinositol 3-kinase, MAP kinase/Erk 1/2 was shown [41]. Finally, a HIF independent but ROS dependent regulation of S100A4 has already been postulated. ROS induces the translocation of phospho-ERK to the nucleus leading to GATA-4 phosphorylation with subsequent S100A4 production [14]. Subsequent interaction with RAGE induces SMC proliferation and migration [14, 42]. In line with this finding, we could observe increased mRNA RAGE expression in the pulmonary arteries of the mouse emphysema model. Although we could not detect differences in RAGE expression between COPD and donors, similar to others [43, 44] we could show RAGE positivity in SMC layer. Of note, systemic soluble RAGE has been suggested as a biomarker as the severity of emphysema was associated with lower levels of sRAGE [45]. Meloche J et al. demonstrated that S100A4 stimulation recapitulated the PAH phenotype of PASMC and that RAGE inhibition attenuated PH in vivo [42]. However, whether the up-regulation of S100A4 in remodeled arteries of COPD lungs is signaled in absence of hypoxia via ROS and HIF has to be determined in further studies.

## Conclusions

We verified a significant up-regulation of S100A4 mRNA level in intrapulmonary vessels of mice after eight month of smoke exposure via laser- microdissection and real-time PCR. On protein level, upregulation of S100A4 was confirmed by semi-quantitative analysis of immunohistochemical labeling. Importantly, we could show an analogous upregulation of S100A4 mRNA and protein in intrapulmonary arteries of COPD-patients. Our results allow us to speculate that a vascular remodeling even in non-hypoxic COPD-stages might involve S100A4. These findings may advance the search for new and effective strategies in the treatment of COPD associated vascular remodeling/pulmonary hypertension.

## Additional files

Additional file 1:
**Supplemental information about data collecion, material and methods.** (PDF 567 kb)

## Abbreviations

COPD: Chronic obstructive pulmonary disease
Egr: Early growth response
EMSA: Electrophoretic mobility shift assay
HIF: Hypoxia inducible factor
HRE: Hypoxia responsive element
PASMC: Pulmonary artery smooth muscle cells
PCR: Polymerase chain reaction
PH: Pulmonary hypertension
RAGE: Receptor for advanced glycation end products
ROS: Reactive oxygen species
RT: Reverse transcription
SMA: Smooth muscle actin
