# Supplementary material for: Increased S100A4 expression in the vasculature of human COPD lungs and murine model of smoke-induced emphysema
Source: Respir Res. 2015 Oct 20;16:127. doi: 10.1186/s12931-015-0284-5 (PMC4612429; doi:10.1186/s12931-015-0284-5)
Supplement: Additional file 1: — Supplemental information about data collecion, material and methods. (PDF 567 kb) [file 12931_2015_284_MOESM1_ESM.pdf]

On-line-Supplement

Gender , age and disease of the applied lung tissue samples

Number	gender	age	disease
1	m	53	Donor
2	w	41	Donor
3	m	53	Donor
4	w	47	Donor
5	m	28	Donor
6	m	57	COPD, GOLD III/IV
7	m	52	COPD, GOLD III/IV
8	w	60	COPD, GOLD III/IV
9	w	53	COPD, GOLD III/IV
10	m	49	COPD, GOLD III/IV

Laser-assisted microdissection

Laser microdissection enables to analyze specific cell types or compartments within complex tissues without contamination. Laser-assisted microdissection was performed using a PALM Laser Microbeam System (Zeiss, Oberkochen, Germany). Cryo-sections (10 µm) from human and mouse lung tissue were mounted on glass slides. Slides were stained in hemalaun for 30 seconds, afterwards dehydrated in an ascending ethanol line (70%, 96% and 100% ethanol) and then stored in 100% ethanol until use. To avoid long storage time, no more than ten sections were prepared at once. A pulsed UV-laser-system (wavelength 337 nm, maximum frequency 20 pulsed per second, pulse duration 3 ns) was used for microdissection. The laser beam was linked to a Zeiss Axiovert 135 inverse microscope with a mechanized object holder and manipulator. Both were computer controlled and allowed an accurate handling of the mechanized tools. Intrapulmonary arteries with a diameter of 50-300 µm were microdissected under optical control. Only endothelial and smooth muscle cell layer of the vessels were selected. Surrounding tissues were removed by laser assisted photolysis. During microdissection slides were covered with 100% ethanol to avoid drying. The vessels were isolated with a sterile 30 G needle and transferred into a reaction tube with RNA lysis buffer (RNeasy kit, Qiagen). 30 vessels were collected into each reaction tube in order to achieve a sufficient number of cells. Tubes were stored at -80°C until use.

Cell lysis and RNA extraction
The RNA from microdissected material was isolated with the RNeasy Micro kit (Qiagen, Hilden, Germany) following the instructions of the user manual with the exception of DNase treatment. For lung homogenate analyses the RNeasy Mini kit (Qiagen) was utilized following the instructions of the user manual including DNase treatment.
Both RNA extraction methods use a spin column technology. Tissue or cell clusters were incubated for 15 minutes in RLT lysis buffer of the RNeasy kit before starting RNA extraction.

First strand cDNA synthesis
Reverse transcription was performed for first strand cDNA synthesis of microdissected human tissues and mouse lung homogenate. For priming random hexanucleotides (PE Applied Biosystems, Weiterstadt, Germany) were used. Each reaction was performed with up to 1 µg of RNA in a final volume of 10 µl and 1 µl of random hexamer primers.

The RNA was denatured at 70°C for 10 minutes and subsequently cooled on ice. The mastermix of following RT-reaction reagents (purchased from PE Applied Biosystems) was added to the reaction and well mixed.

Components	Volume (µl)
RT 10x Buffer II	2
25 mM $MgCl_2$	4
dNTP mix 10 mM	1
RNAse inhibitor 2000 units	0.5
MuLV reverse transcriptase 5000 units	0.5
RNAse free Water	0.5

The mix was incubated in a thermo-cycler at 20°C for 10 minutes to allow annealing of random hexamer primers. The RT-reaction was performed at 43°C for 75 minutes and stopped by heating for 5 minutes at 70°C. Afterwards, the cDNA was cooled on ice and further used for real-time PCR or stored at -20°C.

Relative mRNA quantification by real-time PCR
Gene regulation was analyzed by quantitative real-time PCR using Δ-Ct method for the calculation of relative changes. Real-time PCR was performed by using the 7900HT Real Time PCR-System (PE Applied Biosystems). The reaction mix (final volume 25 µl), and the cycling protocol are given in the table below.

Real time-PCR mastermix

Components	Concentration	Volume (µl)	Final concentration
SYBR Green qPCR SuperMix (Invitrogen)	2x	13	1x
$MgCl_2$	50 mM	1	2 mM
forward primer	1 µM	0.5	200 nM
reverse primer	1 µM	0.5	200 nM

RNAse free Water	8
cDNA	2

Human and mouse primers were validated previously. Primer sequences, amplicon sizes and Accession numbers are given.

Gene	Species	Gene Bank Accession Nb.	Primer Sequence (5` → 3`)		Amplicon Length [bp]
			Forward	Reverse	
PBGD	Mouse	M28664	GGTACAAGGCTTTCAGCATCGC	ATGTCCGGTAACGGCGGC	135
B2M	Mouse	AK019389	AGCCCAAGACCGTCTACTGG	TTCTTTCTGCGTGCATAAATTG	128
S100A4	Mouse	D00208	AGGAGCTACTGACCAGGGAGCT	TCATTGTCCCTGTTGCTGTCC	103
PBGD	Human	NM000190	TGTCTGGTAACGGCAATGCG	CCCACGCGAATCACTCTCAT	70
S100A4	Human	M80563	CTA AAGGAGCTGCTGACCCG	TCCCTGTTGCTGTCCAAGTTG	95
RAGE	Human	NM001136	GCCACTGGTGCTGAAGTGTA	TCCGGCCTGTGTTCAGTTTC	76
B2M	Human	NM004048	TGGAGGCTATCCAGCGTACT	TGTCGGATGGATGAAACCCA	110

The real-time PCR was performed for 45 cycles as follows:

Real time-PCR cycling protocol

Step	Temperatur	Time
Denaturation	95°C	5 min
45X		
Denaturation	95°C	5 s
Annealing	60°C	5 s
Extension	72°C	10 s

Due to the non-selective dsDNA binding of the SYBR Green I dye, melting curve analysis and gel electrophoresis were performed to confirm the exclusive amplification of the expected PCR product.
Two different reference genes (HMBS and β_2 microglobolin) were used for internal control.

For relative mRNA quantification of human tissue and lung homogenate *HMBS* was used, whereas results from laser microdessected vessels of smoke exposed mice were normalized to the relative expression of β_2 microglobulin gene. The relative amount of the target gene is expressed in ΔC_t values (C_t=C_t reference - C_t target).

Immunohistochemistry

For cryo-sections an APAAP (alkaline phosphatase anti alkaline phosphatase)-conjugated antibody system was applied. For color reaction the complex bound alkaline phosphatase reacts with enzyme substrates to alter them into colored particles. A bridge antibody from a second species with a specificity against the primary antibody and the immunoglobulin incorporated into the APAAP complex was utilized to enable specific labeling.
Peroxidase conjugated ImmPress Universal Antibody Kit was utilized for paraffin

sections. In contrast to the APAAP system the ImmPress staining system uses “micro-polymers“ that are directly conjugated to the secondary antibody. “Micro-polymers“ are polymerized peroxidase enzymes that allow a high density of active enzymes to be conjugated directly to the secondary antibody.
Antibodies that are utilized in the study are shown in tabular below.

Alkaline phosphatase conjugated Anti-goat antibody	Rockland, Gilbertsville, USA
Goat-anti-rabbit IgG	Southern Biotech, Eching, Germany
ImmPress Universal Antibody Kit	Vector laboratories, Burlington, Canada
ImmPress Anti Rabbit Ig-Antibody kit	Vector laboratories, Burlington, Canada
Rabbit polyclonal S100A4 antibody	Abcam, Cambridge, UK
SMC α-actin antibody rabbit	NeoMarkers, Fermont, USA

Immunohistochemistry of murine tissue

Cryo-sections were assembled utilizing a -20°C cooled cryotome. Lung tissue was cut at 10 µm and mounted on Superfrost glass slides. Slides were dried over night and stored at -20°C until use. They were fixed in acetone for 10 minutes, subsequently put in 0.1% Triton X100 for 15 minutes and finally washed twice in Tris buffered saline. All antibodies were diluted in Dako Real Antibody Diluent (Dako, Glostrup, Denmark). Rabbit polyclonal S100A4 antibody was used as primary antibody with a 1:500 dilution. Slides were incubated with the primary antibody for one hour and were washed again. Subsequently, the slides were incubated with the secondary antibody goat anti-rabbit IgG in a 1:150 dilution for 45 minutes. After washing, alkaline phosphatase conjugated anti-goat antibody in a 1:200 dilution was applied for 45 minutes. For labeling, the slides were incubated for 30 min in the APAAP substrate solution. Negative controls were prepared with omission of the first antibody. For SMC labeling rabbit polyclonal SMC α-actin antibody in a 1:350 dilution in 10% BSA was applied for 30 minutes. After washing, the slides were developed with the NovaRED substrate kit (Vector laboratories, Burlington, Canada) for 10 minutes and counterstained with hemalaun.

APAAP substrate solution

The APAAP substrate solution was prepared by adding 25 ml propandiol to 70 ml of developing buffer stock, which was composed of 87 g sodium-chloride, 15 g TRIS HCl, 49 g TRIS base and 1750 ml deionized water. The blend was adjusted to pH 9.75 before adding 40 mg of levamisol. Further, 20 mg of sodium-nitrit solved in 500 µl distillated water and 200 µl of new- fuchsin were added. The solutions were mixed and 50 mg naphthol, solved in 600 µl N,N-dimetylformamid, was added to the blend. The working solution was adjusted to pH 8.8 and subsequently filtrated to remove big color particles.

Immunohistochemistry of human-tissues

Paraffin-sections (2 µm thick) from human lung tissue were mounted on Superfrost

glass slides. The slides were dried over night at 37°C and then stored at room temperature. They were deparaffinized by heating at 59°C for one hour, washed three times in xylol for 10 minutes and afterwards rehydrated in descending ethanol line (100%, 96% and 70% ethanol). To avoid non-specific staining, endogenous enzymatic activity of slides was blocked by incubating them for 20 minutes in 15% hydrogen peroxide. Afterwards slides were washed in water and phosphate buffered saline (PBS). All slides were treated with 50% trypsin dilution at 37°C for 15 minutes. After washing in PBS, the sections were blocked for 1 hour with 10% BSA protein block, and for 20 minutes with normal horse serum (ImmPress Universal Antibody kit). For S100A4 labeling, rabbit polyclonal S100A4 antibody at 1:700 dilution in 10% BSA was applied. For SMC labeling, rabbit polyclonal SMC α-actin antibody at 1:350 dilution in 10% BSA was used. Incubation was done over night. The slides for S100A4 labeling were washed and incubated with the peroxidase conjugated secondary antibody (ImmPress Universal Antibody kit) for 30 minutes. For SMC labeling, ImmPress Anti-Rabbit-Ig Antibody kit, (Vector laboratories) was used. The slides were developed with the NovaRED Substrate Kit and counterstained for 10 minutes with hemalaun after washing.

Semi-quantitative analysis of immunolabeled tissue sections
Microscope pictures were taken by the Discus software. Standard magnification was 200x for cryo sections and 100x for paraffin sections. To ensure representative measurement, ten vessels with a diameter between 25-250 µm from each animal were randomly selected. Images were stored in TIF files and analyzed for labeling intensity via Adobe Photoshop software. Average pixel intensities of the magenta channel were determined for the tunica media of the vessels by lightness measurement of the selected areas. To analyze the magenta channel, CMYK color profile was chosen for all pictures. Further cyan was subtracted from the whole image to avoid cell core interference. To accentuate the lightness of the specific staining, the color profile of magenta was added via mask-function of the newly generated cyan free channel. Mean lightness-intensity of the selected area was identified by histogram. To avoid measurement of non-specific staining, by hemalaun cell core staining or unspecific binding of the secondary antibody, intensity of the IHC-stained slides had to be corrected by subtracting staining intensity of the respective negative control.

Electrophoretic mobility shift assay (EMSA)
EMSA was performed with LightShift Chemiluminescent EMSA Kit (Pierce, Rockford, USA) following the instructions of the user manual. Aberrant to the manual the 20 µl reaction mixtures contained 2 µl of 10x binding buffer, 50 ng poly (dI-dC), 1 µl 50% glycerol, 1 µl 1% NP-40 and 5 mM $MgCl_2$. Depending on the experiment 20 fmol biotin labeled probes and 4 pmol unlabeled probes were added (see Table below). The probes were annealed following the Pierce tech tip 45 option 1. In all assays 20 µg of nuclear extract was used. Polyacrylamid gel was prepared by mixing 19.5 ml of distillated water, 1.25 ml of 10x TBE-buffer, 4.2 ml of 30% polyacrylamide, 125 µl of 10% APS and 12.5 µl TEMED. DNA-Crosslink was performed for 10 minutes with a UV lamp equipped with 254 nm bulbs at a distance of approximately 0.5 cm from the membrane.
Sequences of EMSA probes and their position are listed below.

**HRE**	**Position**	**Primer Sequence (5` → 3`)**	
		**Sense**	**Antisense**
HRE 1	-4694	GGCTTAGCCACGTGCTCTCCAAGTTCTAC	GTAGAACTTGGAGAGCACGTGGCTAAGCC
HRE 2	857	CGTGAGCCCCCACGCCTCACTCACTGCCACTC	GAGTGGCAGTGAGTGAGGCGTGGGGGCTCACG
HRE 3	1359	CCAGCAGGAGGGCGTGGGGACAAAAC	GTTTTGTCCCCACGCCCTCCTGCTGG
